# Short sleep time may be the main reason for irregular breakfast to cause overweight—a cross-sectional study

**DOI:** 10.3389/fnut.2024.1310155

**Published:** 2024-01-17

**Authors:** Wei Yang, Zhao Zhuang, Pengxiang Huang, Man Zhang, Kebo Wang, Ying Jiang, Han Zhou, Lianlong Yu

**Affiliations:** ^1^Shandong Provincial Third Hospital, Jinan, Shandong, China; ^2^Qingdao Central Hospital, Qingdao, Shandong, China; ^3^Shandong Center for Disease Control and Prevention, Jinan, Shandong, China

**Keywords:** students, overweight, sleep time, breakfast, cross-sectional study

## Abstract

**Introduction:**

In recent years, the relationship between circadian rhythm and overweight and obesity has attracted the attention of many scholars.

**Methods:**

To evaluate association between the duration of sleep and the regularity of breakfast and overweight. A total of 1,178 students from Qingdao University were selected by stratified cluster sampling. There were 601 males (24.69 ± 0.80 years old) and 569 females (24.54 ± 0.70 years old). We used body mass index (BMI), waist circumference (WC), and waist-to-hip ratio (WHR) to define overweight levels. Chi-square test, Pearson correlation test, and logistic regression were applied to test association among overweight, sleep duration, sleep onset time, and breakfast regularity. Pittsburgh sleep quality index was used to assess the overall sleep quality of the study subjects. Mediation effect and Sobel test were used to analyze the effect of sleep duration on breakfast regularity and overweight.

**Results:**

Only 34.1% of the population ate breakfast every day, and eating breakfast 1–3 times per week was associated with a higher risk of overweight (BMI: OR = 2.183, 95%CI: 1.369,3,481; WC: OR = 2.101, 95%CI: 1.232,3,583; WHR: OR = 2.108, 95%CI: 1.331,3,337). The effects of all types of Usual Breakfast Consumption Frequency on overweight were fully mediated by sleep duration (*p* < 0.05). In particular, the subjects exercised outdoors more than five times per week slept longer (*p* < 0.05).

**Conclusion:**

Short sleep duration may be the main reason for irregular breakfast leading to overweight. Adequate outdoor exercise is essential for weight maintenance.

## 1 Introduction

Debate continues over the optimal number and timing of eating episodes for overweight individuals. The results of a meta-analysis showed that (1) skipping breakfast increases the risk of overweight, and these results are consistent across age, sex, region, and economic conditions. Further research has also found that (2) eating breakfast irregularly may be detrimental to weight status. This may be related to changes in energy intake due to dietary intake. Studies have shown that the presence or absence of energy intake and the duration and regularity of energy intake may change energy balance to varying degrees (3), including by affecting fat storage and insulin sensitivity. And the change of energy balance is one of the causes of overweight (4). At the same time, different forms of energy expenditure such as exercise may also affect the energy balance through energy expenditure.

The relationship between dietary intake and energy balance may be related to circadian rhythms (5). The circadian system represents all physiological processes involved in the 24-h cycle, such as sleep/wake cycles, blood pressure, heart rate, hormone secretion, cognitive performance, and emotion regulation (6). Research shows that (7) SIRT1 gene is associated with circadian rhythm, and the activity of protein can be regulated through BMAL1 dysplasia process to influence the lipid metabolism and sugar. At the same time, circadian dysrhythmization increases postprandial ghrelin levels and decreases leptin levels, leading to the risk of weight gain (8). The duration of feeding is closely related to the expression of genes involved in circadian rhythm, and irregular eating can also affect the stability of diet-related clock oscillators located in peripheral tissues of the central nervous system (9). Sleep is an important component of the circadian rhythm, and different scholars have reached different conclusions in overweight/obesity-related studies. Spruyt et al. (10) showed a positive association between irregular sleep and overweight irrespective of sleep duration. Short sleep duration is a risk factor for overweight in a cohort study of type 2 diabetes mellitus (11).

It can be concluded that both dietary intake and sleep affect circadian rhythm adjustment. And overweight is one possible consequence of the altered circadian rhythms. Most previous studies have focused on the single effects of dietary intake and sleep on overweight, and few have included physical activity factors, which may also affect circadian rhythms. Considering that breakfast is the first meal after a long fast compared to other meal times, it may be particularly important in influencing circadian rhythms (2). Compared with students at other stages, graduate students have more academic pressure and mental pressure, so the overwork, irregular work and rest, obesity, sleep disorders, psychological problems of graduate students are more prominent. Therefore, this study aimed to explore the mediating role of sleep duration in the association between irregular breakfast intake and overweight and obesity in postgraduates. We used different methods to define overweight, and the results were stratified according to frequency of outdoor activity.

## 2 Materials and methods

### 2.1 Study population

**Figure 1:**
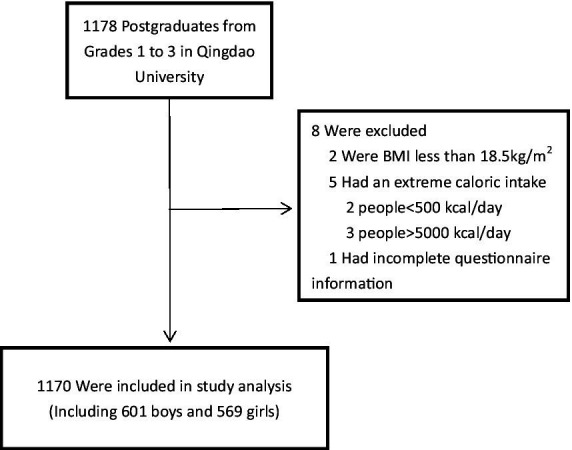
Study population.

A stratified cluster sampling method was used in this study. Specifically, all graduate students in Qingdao University were stratified according to grade, and 8 classes were randomly selected from each class, and all students in the whole class were selected as the research objects. During the research process, we sought the opinions of the school, and after obtaining the consent, we contacted the class teachers and student representatives. After obtaining their cooperation, we summoned all the students in the sampled classes to publicize our research project to the students. Finally, according to the arrangement of the head teacher and the student representative, the questionnaire collection was completed in batches. A total of 1,178 students including 571 females and 602 males were enrolled in this study. After obtaining informed consent, a face-to-face questionnaire was completed to collect anthropometric indicators (It is filled in by the students themselves) and basic information forms, including a lifestyle and physical activity questionnaire (This study was approved by the Ethics Committee of the Third Hospital of Shandong Province and the Affiliated Hospital of Qingdao University with the approval numbers of KYLL-2023085 and QYFYWZLL25548, respectively). In the present analysis, participants were excluded if they had an extreme body mass index (BMI, BMI = weight (kg)/height^2^ (m); below 18.5 kg/m^2^, and above 28.0 kg/m^2^; *n* = 2). Or if they had an extreme caloric intake (<500 or > 5,000 kcal/day; *n* = 5) (12). The effective sample size was 1,170, consisting of 601 boys and 569 girls (Figure 1).

### 2.2 Diet, sleep, and physical activity

An adjusted version of the standard food frequency questionnaire (FFQ) was administered to evaluate dietary intakes. The questionnaire included eight main food groups and food varieties adapted to the Chinese diet. Participants were asked, “During the past year, on average, daily, weekly, or monthly use condition, and average consumption per serving (g)” A similar question asked everyone. Food models and food maps were used to assist in the completion of dietary questionnaires. Average daily energy intake levels for each individual were obtained from the FFQ. The eating habits questionnaire included the eating time of three meals a day, the duration of each meal, whether to eat midnight snacks after dinner, and whether to watch videos during meals. For breakfast, we designed the type of breakfast, how much money to buy breakfast, where to eat breakfast and whether parents value breakfast. Of these, breakfast was defined as eating before 9 a.m. Late-night snacks were defined as meals eaten after dinner in the evening, and any food consumed after dinner was considered a late-night snack.

The Pittsburgh sleep quality index (PSQI) (13) was used to assess the overall sleep quality of the study subjects. It was developed in 1989 by Buysse et al. (14), a psychiatrist at the University of Pittsburgh, USA, to evaluate the sleep quality of the subjects in the last month. It is by far the most widely used sleep quality assessment tool (15). It consists of 18 items consisting of 7 components, each of which is scored on a scale of 0–3, and the cumulative score of each component is the total PSQI score. The total score ranged from 0 to 21, with higher scores indicating poorer sleep quality. A score of 0–5 is defined as very good sleep quality, 6–10 as good sleep quality, 11–15 as fair sleep quality and 16–21 as very poor sleep quality. The questionnaire recorded the time of getting up in the morning and the time of going to bed the previous day, and we used this data to calculate the time in bed each day, subtracting the “time needed to fall asleep” to obtain the actual amount of sleep each day. Participants were told that the duration of sleep did not include periods of lying awake in bed.

The physical activity questionnaire included the number of outdoor activities per week, the time of each activity, etc. Among them, effective outdoor activity was defined as spontaneous physical activity of moderate to vigorous intensity that required moderate to substantial effort and resulted in significantly increased heart rate or shortness of breath. Such as fast walking, dancing, running, climbing, swimming, skipping rope, football, volleyball, basketball and so on.

### 2.3 Adiposity outcomes

Overweight or Central obesity status was defined in cross-sectional analyses using BMI, waist circumference (WC) and waist-to-hip ratio (WHR, WHR = waist circumference/hip circumference). For data collection, height and weight were self-reported by students. Waist and hip circumference were obtained at the physical examination center with the consent of schools, teachers and students themselves. BMI was used to assess both normal (18.5–24.0 kg/m^2^) and obesity (≥24.0 kg/m^2^) as binary outcomes. The definitions of overweight by waist circumference and waist-to-hip ratio refer to previous literature and are adjusted according to the actual situation in China (16, 17). In the separate models, people were considered obese if they had a WC greater than 85 cm for men or 90 cm for women. For WHR, obesity was defined as greater than 0.9 in men and 0.8 in women. We mentioned in the study of obesity are judged to be in the center of the “overweight” in the crowd, the proportion of central obesity.

### 2.4 Statistical analysis

**Figure 2:**
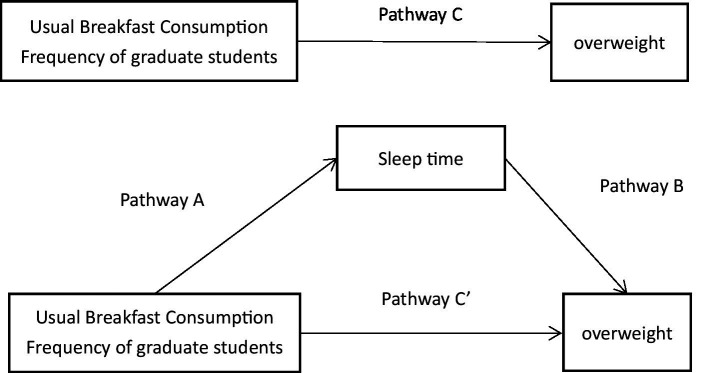
Model of the potential mediating effect of sleep duration on the relationship between usual breakfast consumption frequency of college students and overweight.

The enumerated data is represented by frequencies and the differences between groups are compared using a chi-square test. We used Pearson correlation and binary logistic regression to analyze the relationship between breakfast consumption regularity and excess weight. In logistics regression, overweight was the dependent variable, breakfast frequency was the independent variable, and the variable was selected as “input.” All the results were adjusted for sex and age. The Chi-square test was used to compare the differences in the proportion of obese people in different breakfast frequency groups. Based on the theory of mediating effects proposed by Baron and Kenny (Causal stepwise regression test) (18–20), sleep duration was considered as a mediator factor (M), usual breakfast consumption frequency (X) was considered as an independent variable, and overweight were considered as a dependent variable (Y) for mediating the effect analysis. The total effect (c) of factor X on factor Y can be decomposed into direct effect (c’) and mediating effect (ab). ‘a’ was the effect of factor X on factor M, and ‘b’ was the effect of factor M on factor Y after adjusting for factor X (Figure 2). The specific steps are as follows: first, analyze the regression of X to Y and test the significance of regression coefficient c; Second, the regression from X to M is analyzed and the significance of the regression coefficient a is tested. Third, we analyze the regression from X to Y with the addition of the mediating variable M, and test the significance of the regression coefficients b and c′. Among them, when both a and b are significant, the significance of c′ is tested to judge the mediation effect; When at least one of a and b is not significant, the Sobel test is used to determine whether a mediation effect is present. Linear regression models were used to analyze the mediation effects, which were subsequently evaluated using the Sobel method. All statistical tests were two-sided at α = 0.05 and all analyses were conducted using SPSS 25.0.

## 3 Results

**Figure 3:**
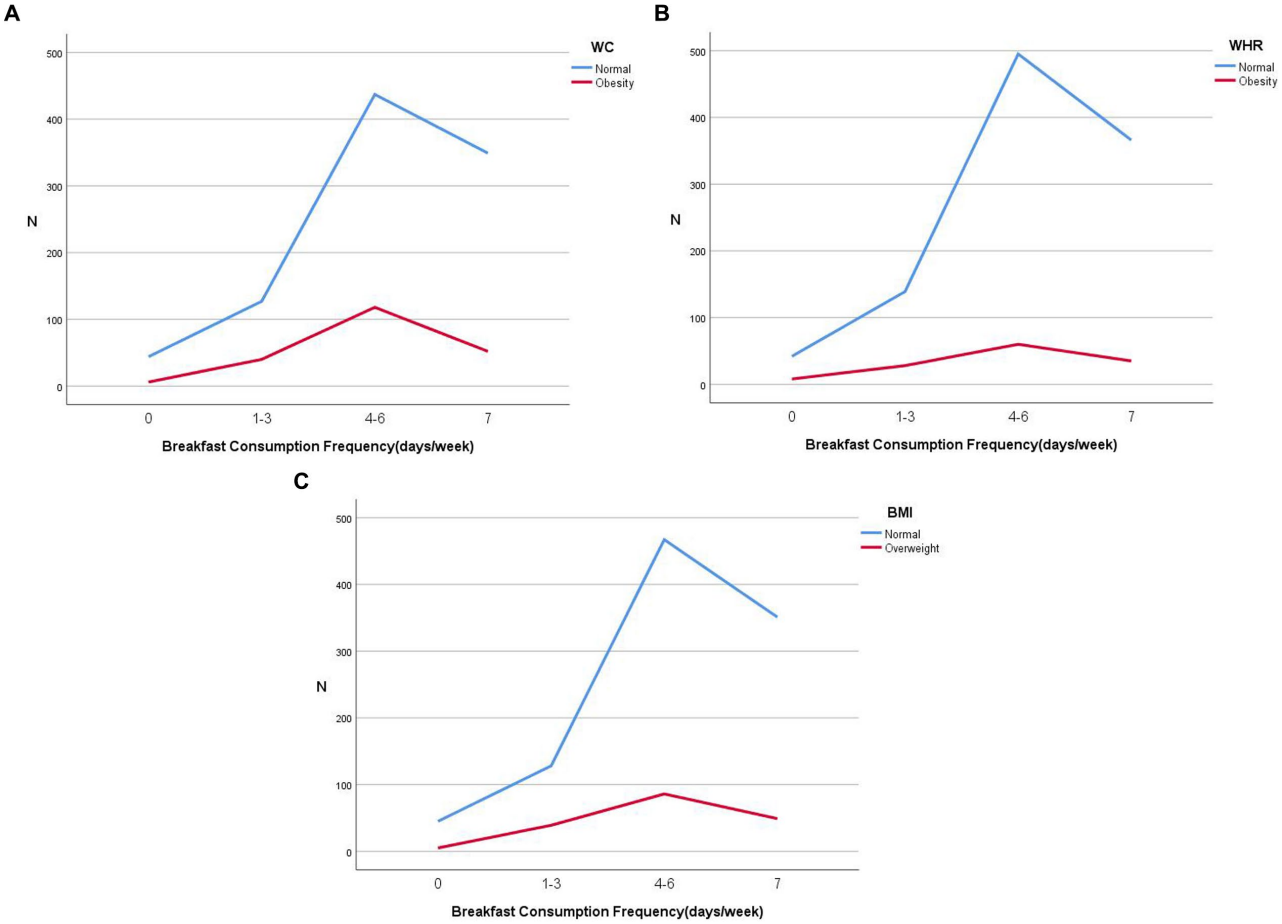
Prevalence of overweight and obesity in different breakfast frequency groups, with overweight defined by waist circumference **(A)**, waist-hip ratio **(B)** and BMI **(C)**, respectively.

The characteristics of the study population, stratified by breakfast consumption frequency, are shown in Table 1. In short, 34.1% of the participants reported eating breakfast daily, 47.3% reported eating breakfast 4–6 days a week and 14.3% reported eating breakfast 1–3 days a week, while only 4.3% of the participants reported not eating breakfast. On average, those who reported eating breakfast daily and 4–6 days a week consumed the most calories, had lower waistlines and WHR, and slept longer. In addition, both those who never ate breakfast and those who ate breakfast daily reported lower BMI than those who ate breakfast 1–3 times a week. Then, those who ate breakfast daily had more weekly outdoor exercise and fewer late-night snacks than the other groups. Shown in Figure 3.

**Table 1:** Characteristics of study population stratified by breakfast consumption frequency.

	Usual breakfast consumption frequency (days/week)			
	0	1–3	4–6	7
*N* (%)	50 (4.3)	167 (14.3)	553 (47.3)	400 (34.1)
Age	24.68 ± 1.05	24.64 ± 0.65	24.63 ± 0.78	24.58 ± 0.74
Gender *n* (%)				
Male	32 (64.0)	93 (55.7)	297 (53.7)	179 (44.7)
Female	18 (36.0)	74 (44.3)	256 (46.3)	221 (55.3)
Energy intake (kcal/day)	1406.73 ± 458.37	1540.70 ± 675.35	1577.29 ± 586.54	1574.58 ± 680.17
WC	76.06 ± 8.5	76.15 ± 10.60	74.87 ± 10.10	74.45 ± 9.02
WHR	0.80 ± 0.07	0.81 ± 0.07	0.79 ± 0.07	0.79 ± 0.06
BMI (kg/m^2^)	21.61 ± 2.76	21.95 ± 2.84	21.60 ± 2.56	21.61 ± 2.44
Average sleep (hours)	6.84 ± 0.90	6.85 ± 0.93	7.00 ± 0.75	7.00 ± 0.82
PSQI score	4.76 ± 2.92	4.93 ± 2.38	4.43 ± 2.34	3.96 ± 2.30
Stay up late (usual sleep after 12 o ‘clock frequency) *n* (%)				
Everyday	13 (26.0)	33 (19.8)	84 (15.2)	42 (10.5)
4–6 times a week	8 (16.0)	37 (22.2)	115 (20.8)	42 (10.5)
1–3 times a week	21 (42.0)	81 (48.5)	286 (51.7)	192 (48.0)
Never	8 (16.0)	16 (9.6)	68 (12.3)	124 (31.0)
Usual outdoor exercise frequency				
≥5 times a week	7 (14.0)	27 (16.2)	86 (15.5)	84 (21.0)
3–4 times a week	16 (32.0)	43 (25.7)	186 (33.7)	140 (35.0)
1–2 times a week	24 (48.0)	92 (55.1)	261 (47.2)	157 (39.3)
Never	3 (6.0)	5 (3.0)	20 (3.6)	19 (4.8)
Late-night snacks frequency				
Everyday	2(4.0)	0	5 (0.9)	2 (0.5)
4–6 times a week	5 (10.0)	19 (11.4)	55 (9.9)	29 (7.2)
1–3 times a month	4 (8.0)	24 (14.4)	63 (11.4)	27 (6.8)
Seldom	23 (46.0)	86 (51.5)	279 (50.5)	195 (48.8)
Never	16 (32.0)	38 (22.8)	151 (27.3)	147 (36.8)

**Figure 4:**
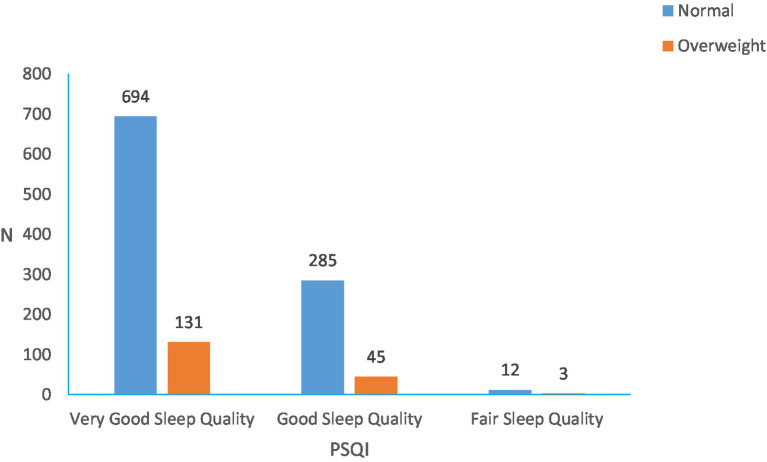
Distribution of overweight and obesity in different PSQI scores (overweight assessed by BMI).

**Figure 5:**
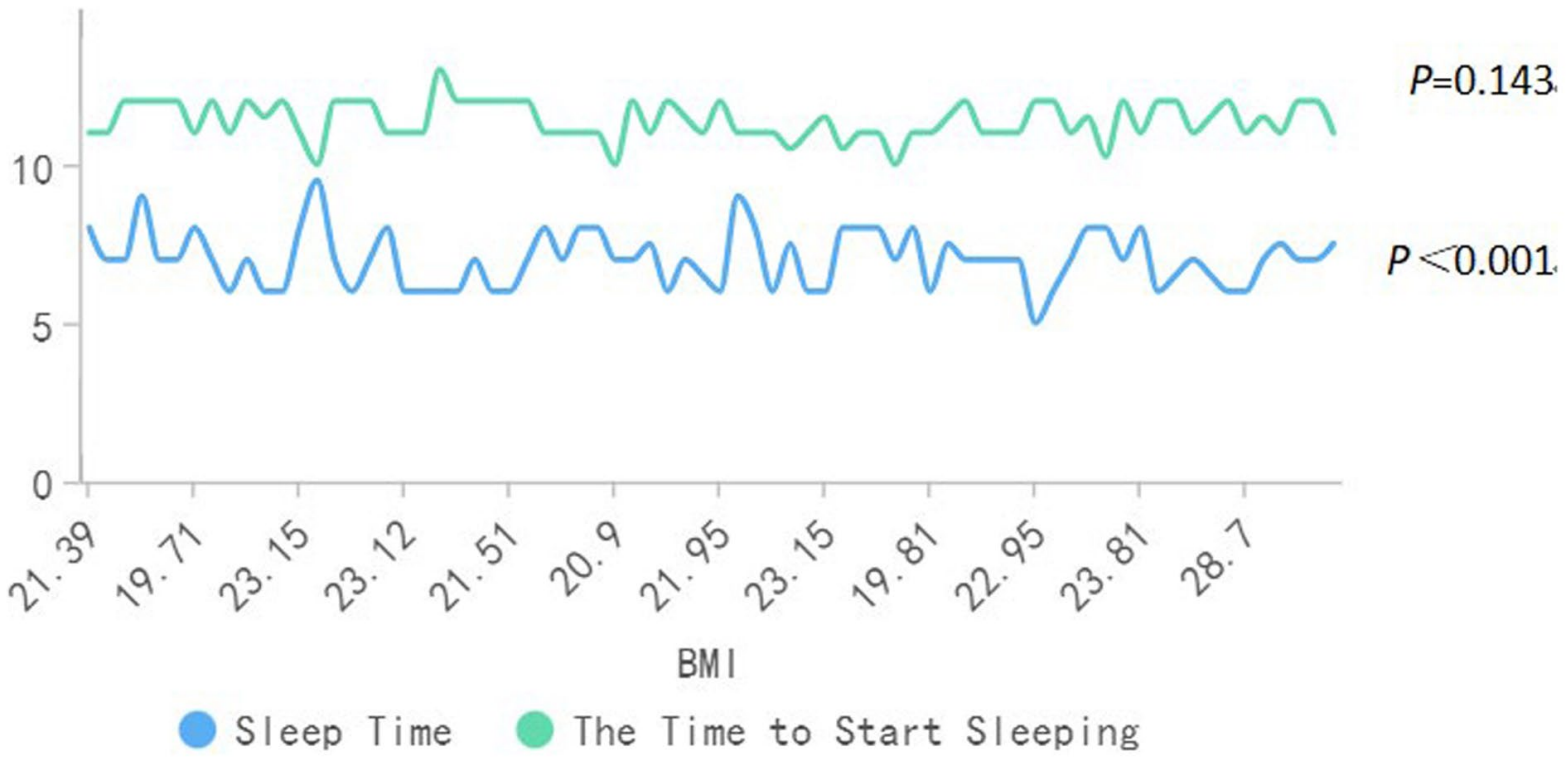
Association between sleep and BMI (In order to let two time be displayed on a diagram, it is expressed in 12 h when I start to sleep. I just started to sleep after the early morning, and it is recorded as the time to start sleeping (12 h) +12).

**Figure 6:**
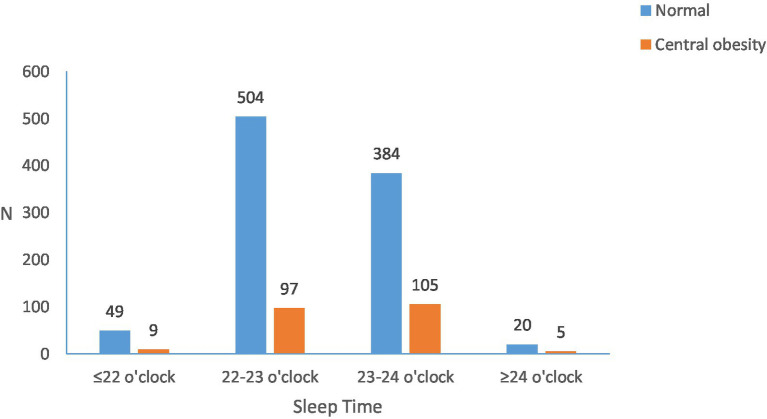
The relationship between the time to sleep and the central obesity.

According to the PSQI scoring rules (Figure 4), 70.5 percent had very good sleep quality, 28.2 percent had good sleep quality and only 1.3 percent had fair sleep quality. The overall PSQI sleep score was 4.35 ± 2.30. The study showed that those who ate breakfast daily had significantly lower PSQI scores than the other groups, with those who ate breakfast 1–3 times a week having the highest mean PSQI scores. We found an association between the duration of sleep and BMI, but not statistically significant between the time of sleep onset and BMI. Shown in Figures 5, 6.

Using BMI to define overweight resulted in the highest prevalence (15.3%), while WHR resulted in the lowest prevalence (11.2%) of obesity, among them, the prevalence of obesity using WC definition was 18.5%. People with different frequency of breakfast intake had different prevalence of overweight, and Pearson’s correlation analysis showed that there was a correlation between overweight and breakfast intake frequency. This association persisted after adjusting for sex and age. In addition, all three overweight determination methods showed the same results (shown in Table 2).

**Table 2:** Relationship between regularity in breakfast consumption and weight status.

		Breakfast consumption frequency (days/week)				χ^2^/p	*p*^a^/*p*^b^
Outdoor exercise frequency		0	1–3	4–6	7		
*BMI (kg/m^2^)*						12.340/0.006	0.039/<0.05
18.5–24	≥5 times a week	4 (2.3)	22 (12.4)	73 (41.2)	78 (44.1)		
	3–4 times a week	15 (4.6)	36 (10.9)	156 (47.4)	122 (37.1)		
	1–2 times a week	23 (5.0)	69 (15.1)	227 (49.7)	138 (30.2)		
	Never	3 (10.7)	1 (3.6)	11 (39.3)	13 (46.4)		
≥24	≥5 times a week	5 (10.0)	39 (23.4)	86 (15.6)	49 (12.3)		
	3–4 times a week	1 (1.8)	7 (12.5)	30 (53.6)	18 (32.1)		
	1–2 times a week	1 (1.3)	23 (29.9)	34 (44.2)	19 (24.7)		
	Never	0	4(21.1)	9(47.4)	6(31.6)		
*WC (Male>85 cm/female>90 cm)*						15.691/0.001	0.020 /<0.05
	≥5 times a week	2 (5.0)	11 (27.5)	19 (47.5)	8 (20.0)		
	3–4 times a week	3 (4.2)	7(9.9)	41 (57.7)	20 (28.2)		
	1–2 times a week	1 (1.1)	18 (20.5)	48 (54.5)	21 (23.9)		
	Never	0	4 (23.5)	10 (58.8)	3 (17.6)		
*WHR(Male>0.9/female>0.8)*						8.930/0.030	0.006/<0.05
	≥5 times a week	8 (16.0)	28 (16.8)	60 (10.8)	35 (8.8)		
	3–4 times a week	3 (7.9)	2 (5.3)	21 (55.3)	12 (31.6)		
	1–2 times a week	3 (4.8)	20 (32.3)	24 (38.7)	15 (24.2)		
	Never	0	1 (14.2)	3 (42.9)	3 (42.9)		

a Pearson correlation.

b Pearson correlation (adjusted for age and sex).

Binary logistic regression analysis was performed with overweight as the dependent variable, breakfast frequency as the independent variable, and daily breakfast frequency as the reference level. When BMI was used to define overweight, the results showed that the likelihood of overweight was 218.3% (OR = 2.183, 95%CI: 1.369, 3.481) for those who ate breakfast 1–3 days per week compared with those who ate breakfast every day. This is similar to the results when the WHR defines overweight (OR = 2.101, 95%CI: 1.232, 3.583). Most associations were observed when using WC to define Central obesity, with those who ate breakfast 1–3 days per week or 4–6 days per week being 110.8% (OR = 2.108, 95%CI: 1.331, 3.337) and 81.5% (OR = 1.815, 95%CI: 1.272, 2.590) more likely to be Central obesity, respectively, compared to people who reported eating breakfast every day. This effect persisted after adjusting for sex and age. The results are shown in Table 3.

**Table 3:** Binary logistics regression analysis of breakfast consumption regularity and overweight in college students under different definitions of overweight.

	Model 1						Model 2	
	Frequency^a^	B	S.E.	Wald	*p*	OR (95%CI)	*p*	OR (95%CI)
BMI	Never	−0.228	0.495	0.212	0.645	0.796 (0.301, 2.102)	0.461	0.692 (0.260, 1.843)
	1–3 times a week	0.780	0.238	10.742	0.001	2.183 (1.369, 3.481)	0.003	2.059 (1.282, 3.308)
	4–6 times a week	0.277	0.192	2.072	0.150	1.319 (0.905, 1.923)	0.254	1.249 (0.852, 1.831)
WC	Never	−0.091	0.460	0.040	0.842	0.913 (0.371, 2.248)	0.636	0.803 (0.324, 1.992)
	1–3 times a week	0.746	0.234	10.113	0.001	2.108 (1.331, 3.337)	0.004	1.986 (1.248, 3.161)
	4–6 times a week	0.596	0.181	10.815	0.001	1.815 (1.272, 2.590)	0.003	1.723 (1.203, 2.467)
WHR	Never	0.686	0.424	2.615	0.106	1.986 (0.865, 4.564)	1.150	1.850 (0.801, 4.270)
	1–3 times a week	0.742	0.272	7.424	0.006	2.101 (1.232, 3.583)	0.010	2.023 (1.183, 3.458)
	4–6 times a week	0.238	0.224	1.136	0.286	1.269 (0.819, 1.967)	0.371	1.223 (0.787, 1.899)

a usual breakfast consumption frequency. And eating breakfast every day was used as a reference.

Model 2: adjusted for age and sex.

We used a causal stepwise regression test to model the mediating effect of sleep duration on the association between frequency of regular breakfast consumption and overweight. We found that sleep duration was associated with the usual breakfast consumption frequency (*p* = 0.015) and overweight (*p* = 0.001). The next test coefficient, c′, was not significant, indicating that sleep duration was a sufficient mediator of the effect of regular breakfast consumption frequency on overweight. We further adjusted for age and gender (model 2) and yielded the same results. The relevant results are shown in Figure 1 and Table 4.

**Table 4:** The mediating effect of sleep duration on the Relationship between usual breakfast consumption frequency of college students and overweight.

	Effect	β	S.E.	*t*	*p*	OR (95%CI)
Model 1	a	0.071	0.029	2.448	0.015	0.014, 0.129
	b	−0.046	0.013	−3.515	0.001	−0.071, −0.020
	C′	−0.053	0.013	−1.826	0.068	−0.050, 0.002
Model 2	a	0.066	0.029	2.265	0.024	0.009, 0.124
	b	−0.092	0.013	−3.173	0.002	−0.067, −0.016
	C′	−0.042	0.013	−1.448	0.148	−0.044, 0.007

All analysis adjusted for gender and ages. a, the effect of usual breakfast consumption frequency of undergraduate and sleep duration; b, the effect of sleep duration and overweight; C′ the direct effect of usual breakfast consumption frequency of undergraduate and overweight.

The mediating effect of sleep duration on the association between frequency of regular breakfast consumption and overweight was stratified by frequency of outdoor activity. Combined with the results of Sobel’s test, when the frequency of outdoor activities was 5 or more times per week, the mediating effect of sleep duration on the association between usual breakfast consumption frequency and overweight existed (*p* = 0.029). The same results were obtained after adjusting for gender and age (*p* = 0.031). The results are shown in Table 5.

**Table 5:** Coefficients for mediation analysis in different outdoor exercise frequency.

Frequency^a^	Direct effect Path C′ β/p	Indirect effect		Sobel test p
		Path A β/p	Path B β/p	
≥5 times a week	−0.082/0.005	0.187/0.008	−0.115/0.101	0.029
3–4 times a week	−0.002/0.933	−0.007/0.890	−0.070/0.522	–
1–2 times a week	−0.025/0.196	0.093/0.053	−0.326/0.001	–
Never	−0.036/0.677	−0.098/0.531	0.011/0.977	–
≥5 times a week^*^	−0.076/0.012	0.187/0.009	−0.117/0.096	0.031
3–4 times a week^*^	0.003/0.908	−0.002/0.960	−0.014/0.548	–
1–2 times a week^*^	−0.021/0.268	0.083/0.060	−0.055/0.003	-
Never^*^	−0.011/0.902	−0.072/0.646	0.011/0.896	–

^a^Different outdoor exercise frequency stratified analyses suggest differences according to outdoor exercise ≥ 5 times a week.

Adjusted for age and sex.

## 4 Discussion

In previous studies (21–23), the prevalence of unhealthy eating habits and overweight among college students has been increasing worldwide. In Canada, the prevalence of overweight was 31.6% in men and 26.6% in women, whereas in a survey of 10,810 people in 12 European countries, almost half of the participants reported being overweight (54.1% in men and 42.5% in women). These are, respectively, higher than the results of the present study. In a study on Shandong Province (China) (24), the prevalence of overweight was higher (22.74% in men and 8.42% in women) and the prevalence of central obesity was lower (7.85% in men and 3.02% in women) than in our study. A study of U.S. found that more than 70% of American adults are overweight or obese (25), which may be related to a dramatic shift in food choices and lifestyles among college students (26). This suggests that overweight among college students remains a social problem that needs to be urgently addressed.

The present study showed that people with regular breakfast consumption habits, including never eating breakfast and eating breakfast every day, had lower mean BMI than those who ate breakfast 1–3 times per week, which is consistent with the results of previous studies (2, 27, 28). In theory, people who eat breakfast every day should have the highest energy intake and correspondingly the highest weight, but in reality they weigh smaller. At the same time, those who ate breakfast daily spent more time outdoors and ate fewer late night snacks per week, suggesting that normal lifestyle habits are beneficial for weight loss (29). Breakfast consumption was sub-divided into four different frequencies in our study, but the average BMI levels associated with eating breakfast daily and eating breakfast 4–6 days per week were similar. We believe that this may be related to the fact that breakfast consumption habits on weekends are different from those on weekdays (30, 31), and whether there is a difference between eating breakfast 6 days a week and eating breakfast every day still needs additional research, and of course it may be related to sampling error.

In this study, 29.5% of the students had poor sleep quality, which was lower than that of Stanford University (42.4%) (32). The overall PSQI score was 4.35, which was similar to the results of South Korea (4.21) (33), lower than the results of Japanese nursing students (6.86) (34), lower than the results of Anhui province (5.37) (35) and Beijing (5.38) (13). In previous studies (36), short sleep duration and unhealthy eating habits were related to overweight, respectively. This study explored the mediating effect of sleep and showed that the duration of sleep is a sufficient mediator of the effect of breakfast consumption on obesity. Sleep duration, as an essential component of the circadian rhythm, may affect fatty metabolism, energy expenditure, and may also have an effect on eating behavior through the circadian rhythm itself. However, the results from a Japanese cohort study showed that (37) skipping breakfast and short sleep time have nothing to do with the prevalence of male obesity, but are related to female obesity. For this result, we still think that there may be differences in sleep habits between men and women. There are also differences in circadian rhythm and energy balance caused by different sleep habits. So whether different sleep habits can change the circadian rhythm of the body is still a problem worth studying.

It has been suggested that (38) staying up late with insomnia may cause adolescents to consume additional carbohydrates and fats, most likely to gain weight. However, our study did not show a statistically significant association between the time of sleep onset and BMI. It could be that the relatively fixed work-and-rest habits of college masked the effects of late nights on weight, or it could be that the effects of staying up for short periods of time on the body’s metabolism have not yet reached the point where they lead to visceral obesity (39). The specific underlying mechanisms require additional investigation.

When the frequency of outdoor activities was ≥5 times per week, the mediating effect of sleep remained significant. In previous studies (40), exercise significantly improved sleep efficiency. Our study may suggest that adequate amounts of exercise are required to have an effective effect on sleep. In fact, numerous scholars also emphasize the importance of regular exercise (41, 42). Regular exercise may improve sleep quality by increasing the production of brain-derived neurotrophic factors (43) and increase skeletal muscle fatty oxidation (44). This may be related to the fact that the effects of exercise on the body’s health are reflected in molecular and cellular changes over a longer period of time (45). This also shows the importance of sticking to exercise and regular lifestyle habits.

There are some limitations in our analysis that should be noted, such as the possible sampling error due to the selection of subjects from only one school. In addition, the strength of our study cancels out the possibility of error due to the small number of people classified as obese. The inclusion of subjects with good homogeneity, similar ages, and consistent living environments also excluded some potential confounding factors for our study.

## 5 Conclusion

In conclusion, this study suggests that short evening sleep duration may be the main cause of overweight caused by irregular breakfast in graduate students. And the mediating effect is still significant when outdoor activities are ≥5 times per week. The differences in the effects of skipping breakfast versus eating breakfast irregularly on obesity are still under debate, and we believe that the effects of breakfast consumption during the week and breakfast consumption during the weekend on obesity remain worthy of study. Sleeping late and sleeping short are two concepts, and the effect of these two different concepts on the body’s metabolism is also a point of concern. Most importantly, this study shows that regular breakfast consumption, adequate sleep duration, and adequate outdoor physical activity are all essential for weight maintenance.

## Data availability statement

The raw data supporting the conclusions of this article will be made available by the authors, without undue reservation.

## Ethics statement

Ethical review and approval was not required for the study on human participants in accordance with the local legislation and institutional requirements. Written informed consent from the patients/ participants or patients/participants legal guardian/next of kin was not required to participate in this study in accordance with the national legislation and the institutional requirements.

## Author contributions

WY: Formal analysis, Investigation, Methodology, Validation, Writing – original draft. ZZ: Investigation, Validation, Writing – review & editing. PH: Investigation, Methodology, Validation, Writing – review & editing. MZ: Investigation, Methodology, Writing – review & editing. KW: Methodology, Writing – review & editing. YJ: Methodology, Writing – review & editing. HZ: Conceptualization, Formal analysis, Investigation, Methodology, Validation, Writing – original draft. LY: Conceptualization, Methodology, Writing – review & editing.
